# Interaction between progesterone exposure duration and blastocyst expansion stage on live birth following frozen–thawed transfer of day-6 blastocysts

**DOI:** 10.3389/fendo.2026.1813202

**Published:** 2026-04-23

**Authors:** Ruiqiong Zhou, Mei Dong, Zhaoyi Wang, Li Huang, Quan Qi, Fuxiang Wang, Liqing Xu, Xiqian Zhang, Fenghua Liu

**Affiliations:** 1 Centre for Reproductive Medicine, Guangdong Women and Children Hospital, Guangzhou, Guangdong, China; 2 Women and Children’s Hospital, Southern University of Science and Technology, Shenzhen, Guangdong, China

**Keywords:** blastocyst expansion stage, day-6 blastocyst, frozen–thawed embryo transfer, live birth rate, progesterone timing

## Abstract

**Objective:**

To determine the optimal duration of progesterone exposure before frozen–thawed embryo transfer (FET) of day-6 blastocysts.

**Methods:**

This retrospective cohort study included 2,058 women who underwent single frozen–thawed day-6 blastocyst transfer between August 2021 and August 2024. Univariable and multivariable logistic regression models were applied to evaluate the association between the duration of progesterone exposure and live birth rate (LBR). Subgroup analyzes with formal interaction testing were conducted to assess potential effect modification by blastocyst expansion stage and other covariates.

**Results:**

Overall LBR was comparable between the day-6 and day-7 progesterone groups (42.8% vs. 41.9%; *P* = 0.691). Crucially, a highly significant interaction was observed between progesterone exposure duration and blastocyst expansion stage (*P* for interaction < 0.01). Among early-stage blastocysts (stages 3-4), LBR was significantly lower following day-7 versus day-6 progesterone exposure (37.3% vs. 46.2%; adjusted OR = 0.70, 95% CI: 0.55-0.87, *P* = 0.002). Conversely, among late-stage blastocysts (stages 5-6), LBR was significantly higher following day-7 versus day-6 exposure (50.2% vs. 36.5%; adjusted OR = 1.82, 95% CI: 1.34-2.48, *P* < 0.001).

**Conclusion:**

While overall LBRs were similar between day-6 and day-7 progesterone regimens for day-6 blastocyst, optimal progesterone duration differed by blastocyst expansion stage: early-stage blastocysts benefited from day-6 exposure, whereas late-stage blastocysts derived benefit from day-7 exposure.

## Introduction

The use of frozen–thawed embryo transfer (FET) has increased substantially worldwide in recent decades, driven by advances in vitrification technology (1). Several protocols have been proposed for endometrial preparation in FET cycles; however, the optimal approach remains uncertain (2, 3). Emerging evidence suggests that artificial FET cycles, which lack a functional corpus luteum, may be associated with a higher risk of adverse obstetric outcomes than natural or stimulated cycles (4, 5). Nevertheless, hormone replacement therapy (HRT) remains widely adopted in clinical practice because of its scheduling flexibility and convenience (2).

Successful implantation and subsequent pregnancy require precise synchronization between embryonic developmental stage and the endometrial window of implantation (WOI) (6). Most existing research has evaluated endometrial receptivity and embryonic potential in isolation; however, implantation failure may still occur if the temporal alignment between these two factors is suboptimal (7). The duration of the WOI remains controversial (8, 9). Earlier studies suggested that embryo implantation can occur over a relatively wide interval following progesterone initiation; however, suboptimal endometrial priming may increase the risk of pregnancy loss (10, 11), highlighting the crucial distinction between achievable and optimal implantation.

In FET cycles, the duration of progesterone exposure prior to embryo transfer is critical for establishing the optimal WOI (10). Nevertheless, the optimal timing of progesterone exposure before embryo transfer—particularly in HRT cycles—remains poorly defined. Several studies have examined the duration of progesterone supplementation before cleavage-stage embryo transfer in FET cycles, with inconsistent findings (12–14). With respect to blastocyst transfer, one randomized controlled trial (RCT) found no significant difference in clinical pregnancy rates between 5-day and 7-day progesterone regimens (15). A retrospective cohort study further reported comparable live birth rates (LBRs) for blastocyst transfers performed on day 6 versus day 7 of progesterone supplementation in artificial FET cycles (16). Notably, in the day-6 blastocyst subgroup, transfers on day 6 were associated with a significantly higher miscarriage rate (50.0% vs. 21.4%), whereas outcomes for day-5 blastocysts were similar between regimens. In clinical practice, a substantial proportion of embryos reach the blastocyst stage on day 6 rather than day 5, with previous studies reporting that approximately 25–45% of blastocysts transferred in FET cycles are day-6 blastocysts (16–18), underscoring the clinical relevance of optimizing transfer strategies for this population.

Embryos exhibiting delayed blastulation have been shown to have lower implantation rates compared with those reaching the blastocyst stage on day 5 (19–23). In addition to the day of blastocyst formation, evidence suggests that the blastocyst expansion stage is also an independent predictor of reproductive outcomes (17, 24). In routine clinical practice, blastocyst transfer—irrespective of developmental timing or expansion stage—is commonly performed after a uniform duration of progesterone exposure, most often six days. Therefore, defining the optimal duration of progesterone exposure for day-6 blastocysts in FET cycles is of substantial clinical importance.

The present study aimed to investigate the optimal timing of progesterone exposure for day-6 blastocysts in artificial FET cycles, with a specific focus on potential differences according to blastocyst expansion stage.

## Methods

### Study design and participants

This retrospective cohort study was approved by the Ethics Committee of Guangdong Women and Children Hospital. Patients undergoing frozen-thawed single-blastocyst transfer cycles with HRT at the Centre for Reproductive Medicine, Guangdong Women and Children Hospital, between August 2021 and August 2024, were included. Exclusion criteria were as follows: age > 40 years at oocyte retrieval; recurrent pregnancy loss (RPL; defined as ≥2 failed clinical pregnancies); repeated implantation failure (RIF); recurrent enrollment; endometrial thickness < 7 mm; uterine abnormalities or intrauterine adhesions; escape ovulation; day-5 blastocyst transfer; and missing core data. RIF was defined as failure to achieve pregnancy after two or more oocyte retrieval cycles with transfer of more than four good-quality cleavage-stage embryos or two good-quality blastocysts. The study was conducted in accordance with the Declaration of Helsinki and adhered to the principles of Good Clinical Practice.

### Endometrial preparation

After confirming that patients were in the early proliferative phase of the menstrual cycle, oral estradiol valerate (Progynova; Bayer Schering Pharma AG, Germany) was started on cycle days 2–4 at 2–3 mg twice daily. After approximately 10 days of estrogen administration, transvaginal ultrasound was performed to evaluate endometrial thickness and morphology, with concurrent measurement of serum estradiol and progesterone levels. The dose and duration of estradiol valerate were adjusted as needed. Once endometrial thickness reached ≥ 7 mm, Progynova was continued, and progesterone supplementation was initiated using either intramuscular progesterone (40 mg once daily) or vaginal progesterone gel (Crinone, Merck Serono, 90 mg once daily) in combination with oral dydrogesterone (10 mg twice daily). The decision to schedule the blastocyst transfer on either the sixth or seventh day of progesterone administration was predominantly determined by non-clinical factors, including individual physician prescription habits and patient convenience. If clinical pregnancy was confirmed, luteal phase support was continued until 10 weeks of gestation.

### Embryos evaluation and transfer procedure

Blastocysts were assessed for expansion, inner cell mass (ICM) quality, and trophectoderm (TE) appearance on day 5 or 6 according to the Gardner and Schoolcraft grading system (25). Embryos graded ≥3BC were cryopreserved. Expansion was classified as follows: stage 3, full blastocyst with the blastocoel completely filling the embryo; stage 4, expanded blastocyst with the blastocoel enlarging the embryo and thinning the zona pellucida; stage 5, hatching blastocyst with the trophectoderm herniating through the zona pellucida; stage 6, fully hatched blastocyst. For primary comparative analyzes, blastocysts were dichotomized into early-stage (stages 3-4) and late-stage (stages 5-6). This classification was theoretically based on the embryo’s morphological status relative to the zona pellucida: stages 3 and 4 remain fully enclosed, whereas stages 5 and 6 are actively herniating or fully hatched, suggesting a potential difference in their immediate readiness for endometrial interaction. Furthermore, this binary grouping ensures adequate sample sizes and statistical power, mitigating the instability of estimates derived from smaller, single-stage subgroups. ICM morphology was graded as A (numerous tightly packed cells), B (several loosely grouped cells), or C (very few cells), and TE morphology was graded as A (many cells forming a cohesive epithelium), B (few cells forming a loose epithelium), or C (very few large cells). Blastocysts were thawed on the morning of embryo transfer, and post-warming survival was defined as ≥50% of blastomeres or blastocyst cells intact without degeneration. Good-quality blastocysts were defined as those with at least stage 3 expansion and both ICM and TE graded A or B (≥3BB). If the first thawed blastocyst did not survive, a second blastocyst was thawed and transferred.

### Outcome measures

The primary outcome was the LBR, defined as the delivery of a live infant at ≥24 weeks of gestation. Secondary outcomes included biochemical pregnancy (serum β-hCG ≥ 10 IU/l at 12–15 days after ET), clinical pregnancy (at least one gestational sac detected by ultrasound at 5 weeks after ET), ectopic pregnancy (extra-uterine pregnancy detected by ultrasound or laparoscopy), biochemical pregnancy loss (initial positive β-hCG that did not progress to clinical pregnancy), clinical pregnancy loss (spontaneous loss of a clinical pregnancy), total pregnancy loss (biochemical and clinical pregnancy loss), and good birth outcome (live birth at 37 weeks or more of gestation, with a birth weight between 2500 and 4000 g and without a major congenital anomaly) (26, 27). Perinatal outcomes included gestational age, cesarean section, preterm birth (< 37 weeks), hypertensive disorders of pregnancy (new-onset hypertension after 20 weeks of gestation in a previously normotensive woman), gestational diabetes (glucose intolerance with onset or first recognition during pregnancy), major congenital anomaly (structural malformations with significant medical, surgical, or cosmetic implications requiring intervention), birthweight, low birthweight (< 2500 g), macrosomia (≥ 4000 g), small for gestational age (birthweight < 10th percentile), large for gestational age (birthweight > 90th percentile), and stillbirth (absence of signs of life at or after delivery).

### Statistical analysis

Normality of continuous variables was assessed using the Kolmogorov–Smirnov test. Continuous variables were expressed as mean ± standard deviation or median with interquartile range, and significant differences were assessed using Student’s t-test or Mann-Whitney U-test, as appropriate. Categorical variables were expressed as frequency with percentage and compared using the χ² test or Fisher’s exact test, as appropriate. Odds ratios (ORs) with 95% confidence intervals (CIs), along with absolute risk differences and their 95% CIs, were reported.

Univariable and multivariable logistic regression analyzes were performed to identify potential confounders independently associated with live birth. Variables with *P* < 0.10 in univariable analysis, or those considered clinically relevant to live birth (e.g., endometrial thickness), were included in the multivariable models. Collinearity among variables was assessed; if two variables were highly correlated, only one was retained in the final model. Model 1 was adjusted for age at retrieval, body mass index, IVF indication, number of IVF cycles, number of oocytes retrieved, preimplantation genetic testing (PGT) cycle, cycle regimen, endometrial thickness, route of progesterone administration, morphological quality grade, blastocyst expansion stage, and duration of progesterone exposure. Model 2 included all covariates from Model 1, with the addition of an interaction term between blastocyst expansion stage and progesterone exposure duration.

Subgroup analyzes were performed according to age (<35 vs. ≥35 years), blastocyst expansion stage (early vs. late), body mass index (BMI) (<24 vs. ≥24 kg/m²), number of IVF cycles (1, 2, or ≥3), cycle regimens (HRT vs. GnRH-agonist HRT), good-quality blastocyst transfer (no vs. yes), and PGT cycle (no vs. yes).

All hypothesis tests were two-sided, with *P* < 0.05 considered statistically significant. Statistical analyzes were performed using SPSS version 22 and R version 4.3.

## Results

### Study population

**Figure 1:**
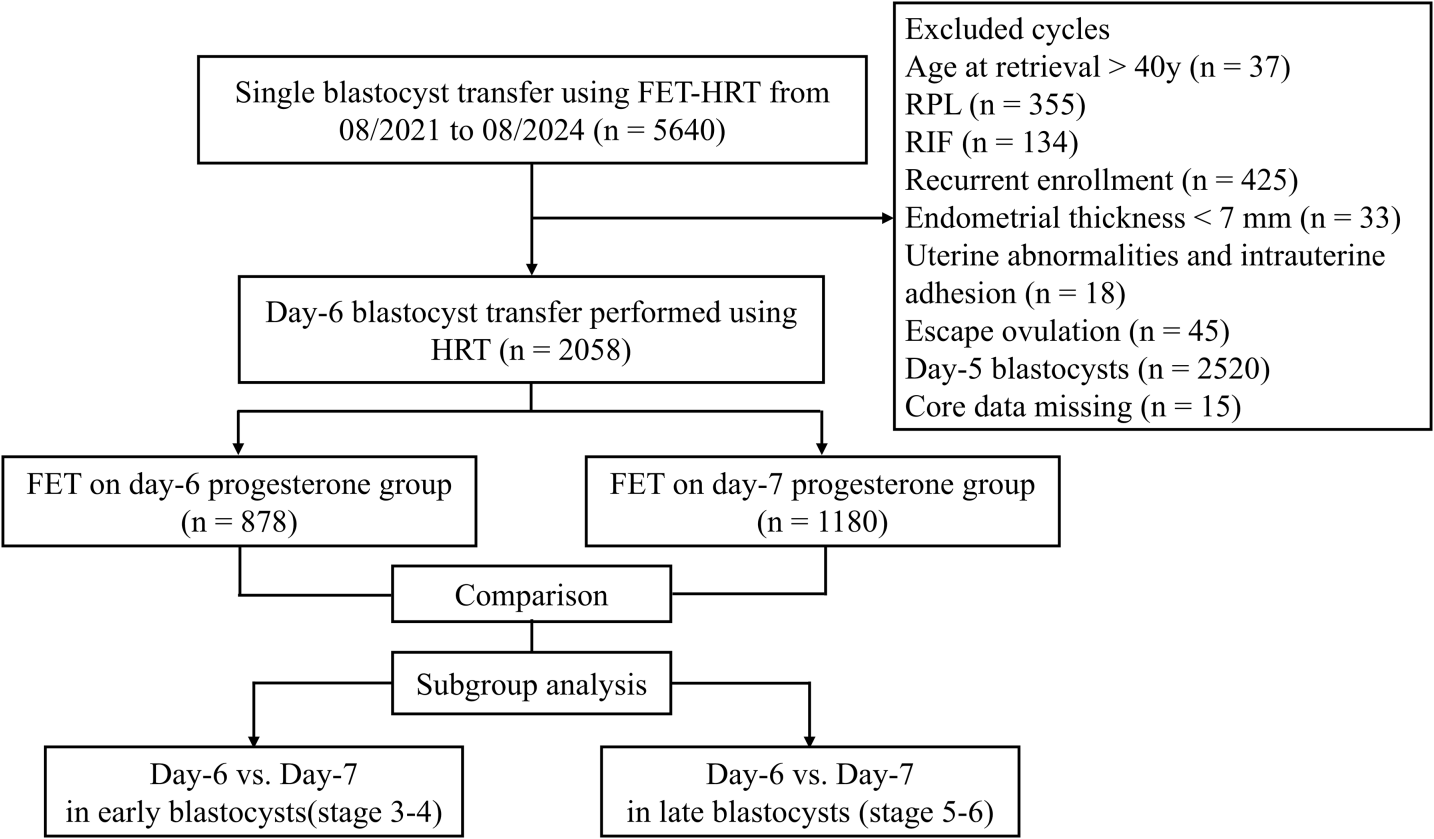
Flowchart of patient enrolment. FET, frozen embryo transfer; HRT, hormone replacement therapy; RPL, recurrent pregnancy loss; RIF, repeated implantation failure.

This study included 2,058 patients who met the inclusion and exclusion criteria (**Figure 1**). The effects of 6 versus 7 days of progesterone administration prior to day-6 blastocyst transfer on clinical outcomes were compared across all patients, with subsequent subgroup analyzes conducted separately for early- and late-stage blastocysts. Baseline characteristics and cycle parameters were well balanced between the day-6 and day-7 progesterone exposure groups in both the overall cohort and the subgroups stratified by blastocyst expansion stage (**Table 1**; **Supplementary Table 1**).

**Table 1:** Baseline demographics and cycle characteristics in day-6 versus day-7 groups.

Parameters	Day-6 progesterone group	Day-7 progesterone group	*P*-value
	(n = 878)	(n = 1180)	
Age at retrieval (years)	31 (28, 35)	32 (28, 35)	0.129
Age at embryo transfer (years)	33 (30, 36)	33 (30, 36)	0.449
Body mass index (kg/m^2^)	21.5 (19.6, 23.7)	21.4 (19.6, 23.4)	0.483
Duration of infertility (years)	3.0 (1.0, 4.0)	3.0 (1.0, 4.5)	0.702
Type of infertility			0.594
Primary	442 (50.3)	580 (49.2)	
Secondary	436 (49.7)	600 (50.8)	
Indications for IVF			0.222
Tubal factor	352 (40.1)	460 (39.0)	
Male factor	142 (16.2)	155 (13.1)	
Others	255 (29.0)	369 (31.3)	
Combined factors	87 (9.9)	126 (10.7)	
Unexplained	42 (4.8)	70 (5.9)	
Antral follicle count	15 (10, 21)	15 (10, 21)	0.897
Number of IVF cycles†			0.714
1	716 (81.5)	972 (82.4)	
2	130 (14.8)	161 (13.6)	
≥3	32 (3.6)	47 (4.0)	
ICSI treatment	414 (47.2)	525 (44.5)	0.231
Number of oocytes retrieved	17 (11, 22)	16 (11, 21)	0.083
PGT cycle	219 (24.9)	305 (25.8)	0.641
PGT method			0.48
PGT-A	74 (33.8)	88 (28.9)	
PGT-M	67 (30.6)	99 (32.5)	
PGT-SR	78 (35.6)	118 (38.7)	
Embryo transfer cycle rank†			0.995
1	332 (37.8)	450 (38.1)	
2	308 (35.1)	408 (34.6)	
3	169 (19.2)	230 (19.5)	
4	69 (7.9)	92 (7.8)	
Endometrial thickness*	9.0 (8.0, 11.0)	9.0 (8.0, 11.0)	0.277
Good-quality blastocyst transfer	477 (54.3)	618 (52.4)	0.379
Morphological quality grades			0.122
Grade 1	67 (7.6)	62 (5.3)	
Grade 2	410 (46.7)	556 (47.1)	
Grade 3	401 (45.7)	562 (47.6)	
Cycle regimens			0.814
HRT	656 (74.7)	887 (75.2)	
GnRH-agonist HRT	222 (25.3)	293 (24.8)	
Route of progesterone supplementation			0.207
Intramuscular + oral	374 (42.6)	470 (39.8)	
Vaginal + oral	504 (57.4)	710 (60.2)	
Blastocyst expansion stage			0.909
3	41 (4.7)	49 (4.2)	
4	530 (60.4)	705 (59.7)	
5	171 (19.5)	235 (19.9)	
6	136 (15.5)	191 (16.2)	

Data are median (IQR) or n (%). IVF, *in-vitro* fertilization; ICSI, intracytoplasmic sperm injection; FET, frozen embryo transfer; PGT, preimplantation genetic testing; A, aneuploidy; M, monogenic disorder; SR, chromosomal structural rearrangement; HRT, hormone replacement therapy. *The day of frozen embryo transfer. †These variables denote the ordinal position of the included cycle. Morphological quality grades are defined by the combination of inner cell mass (ICM) and trophectoderm (TE) scores: Grade 1 (AA, AB, BA); Grade 2 (BB); and Grade 3 (BC, CB, AC, CA).

### Pregnancy and perinatal outcomes

Pregnancy and perinatal outcomes are listed in **Table 2**. The LBR was 42.8% in the day-6 progesterone group and 41.9% in the day-7 group (absolute difference, –0.88% [95% CI, –5.19% to 3.44%]; OR, 0.97 [95% CI, 0.81–1.15]; *P* = 0.691). Rates of biochemical pregnancy, clinical pregnancy, pregnancy loss, ectopic pregnancy and good birth outcome were similar between the two groups. Obstetric and perinatal outcomes were also comparable (**Table 2**).

**Table 2:** Pregnancy and perinatal outcomes in day-6 versus day-7 groups.

Outcomes	Day-6 progesterone group (n=878)	Day-7 progesterone group (n=1180)	Absolute difference	Odds ratio	*P* value
			(95% CI)	(95% CI)	
Live birth	376/878 (42.8)	495/1180 (41.9)	-0.88 (-5.19 to 3.44)	0.97 (0.81 to 1.15)	0.691
Biochemical pregnancy	550/878 (62.6)	710/1180 (60.2)	-2.47 (-6.72 to 1.77)	0.90 (0.75 to 1.08)	0.255
Clinical pregnancy	478/878 (54.4)	624/1180 (52.9)	-1.56 (-5.92 to 2.79)	0.94 (0.79 to 1.12)	0.483
Total pregnancy loss	165/550 (30.0)	209/710 (29.4)	-0.56 (-5.65 to 4.53)	0.97 (0.76 to 1.24)	0.828
Biochemical pregnancy loss	72/550 (13.1)	86/710 (12.1)	-0.98 (-4.68 to 2.72)	0.92 (0.66 to 1.28)	0.603
Clinical pregnancy loss	93/478 (19.5)	123/624 (19.7)	0.26 (-4.47 to 4.98)	1.02 (0.75 to 1.37)	0.916
Ectopic pregnancy	6/478 (1.3)	4/624 (0.6)	-0.61 (-1.79 to 0.56)	0.51 (0.14 to 1.81)	0.345
Good birth outcome	316/878 (36.0)	408/1180 (34.6)	-1.41 (-5.59 to 2.76)	0.94 (0.78 to 1.13)	0.506
Gestational age*	38.1 (1.7)	38.2 (1.8)	0.1 (-0.1 to 0.3)		0.726
Preterm birth*	39/376 (10.4)	62/495 (12.5)	2.15 (-2.09 to 6.40)	1.24 (0.81 to 1.89)	0.326
Cesarean section*	257/376 (68.4)	342/495 (69.1)	0.74 (-5.48 to 6.96)	1.04 (0.78 to 1.38)	0.815
Hypertensive disorders of pregnancy*	28/376 (7.4)	38/495 (7.7)	0.23 (-3.31 to 3.77)	1.03 (0.62 to 1.72)	0.899
Gestational diabetes*	101/376 (26.9)	135/495 (27.3)	0.41 (-5.54 to 6.37)	1.02 (0.76 to 1.38)	0.892
Major congenital anomaly*	2/376 (0.5)	6/495 (1.2)	0.68 (-0.53 to 1.89)	2.29 (0.46 to 11.43)	0.477
Birthweight (g)†	3172 (522)	3161 (507)	-11 (-80.3 to 58.3)		0.747
Low birthweight†	27/375 (7.2)	40/493 (8.1)	0.91 (-2.64 to 4.47)	1.14 (0.69 to 1.89)	0.617
Macrosomia†	13/375 (3.5)	16/493 (3.2)	-0.22 (-2.65 to 2.20)	0.93 (0.44 to 1.97)	0.857
Small for gestational age†	27/375 (7.2)	51/492 (10.4)	3.17 (-0.59 to 6.92)	1.49 (0.92 to 2.43)	0.107
Large for gestational age†	33/375 (8.8)	47/492 (9.6)	0.75 (-3.12 to 4.62)	1.10 (0.69 to 1.75)	0.704
Stillbirth	2/378 (0.5)	0/495	-0.53 (-1.26 to 0.20)		0.187

Data are n (%) or mean (SD). *Among live newborns. †Among all singletons.

Univariable and multivariable logistic regression analyzes were performed to evaluate factors associated with live birth in the overall cohort, adjusting for potential confounders (**Supplementary Tables 3**, **4**). In Model 1, duration of progesterone exposure prior to embryo transfer was not independently associated with live birth (adjusted OR = 0.99; 95% CI, 0.82–1.18; **Supplementary Table 4**). Model 2 included all covariates from Model 1 plus an interaction term between blastocyst expansion stage and progesterone exposure duration. In Model 2, the effects of day-7 (vs. day-6) progesterone exposure (adjusted OR = 0.70; 95% CI, 0.56-0.88; *P* = 0.002) and late-stage (vs. early-stage) blastocysts (adjusted OR = 0.61; 95% CI, 0.46-0.82; *P* = 0.001) were both statistically significant. Crucially, the interaction term was highly significant (adjusted OR = 2.58; 95% CI, 1.76-3.77; *P* < 0.001), confirming that the optimal duration of progesterone exposure fundamentally differs according to the expansion stage. The full output of Model 2 is detailed in **Supplementary Table 4**.

### Subgroup analyzes

**Figure 2:**
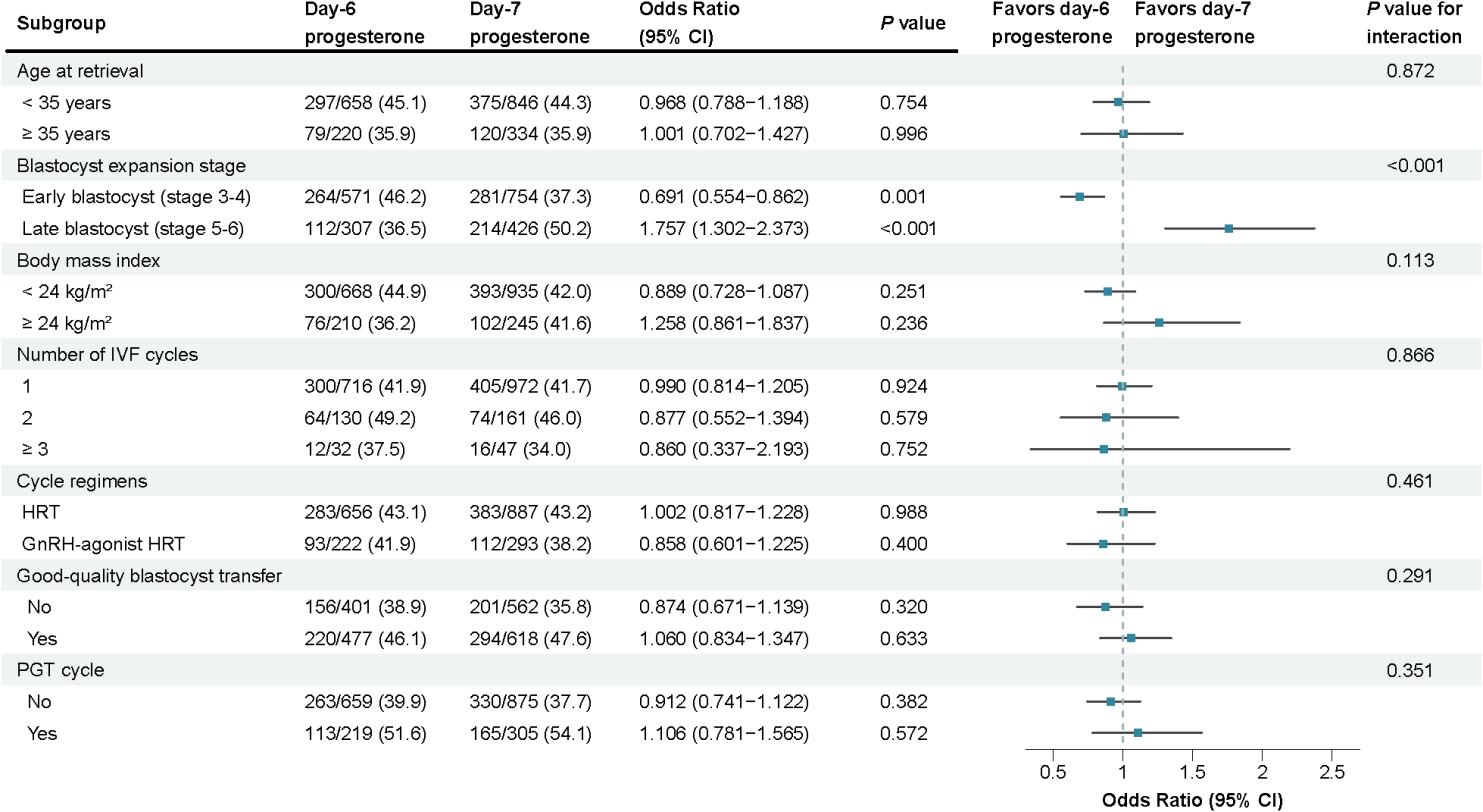
Subgroup analyzes for live birth rate.

Subgroup analyzes were conducted by age, blastocyst expansion stage, BMI, type of infertility, number of IVF cycles, good-quality blastocyst transfer, and PGT cycle (**Figure 2**). No significant interactions were observed between progesterone exposure duration and any factor with respect to live birth rate, except for blastocyst expansion stage. A significant interaction was detected between blastocyst expansion stage and progesterone exposure group (*P* for interaction < 0.01; **Figure 2**), with consistent results observed when stratified by four expansion stages (*P* for interaction < 0.01; **Supplementary Table 2**). However, it is important to note that the sample sizes within the single-stage subcategories (e.g., stage 3 with day-7 progesterone, n=49) are relatively small. This inherently limits the statistical power, resulting in wider confidence intervals for these specific point estimates, which should therefore be interpreted with caution.

Among women undergoing early blastocyst transfers (stage 3–4), the live birth rate was significantly lower in the day-7 progesterone group than in the day-6 group (37.3% vs. 46.2%; absolute difference, –8.97%; 95% CI, –14.32% to -3.62%; *P* = 0.001) (**Table 3**). Similarly, rates of biochemical pregnancy, clinical pregnancy, and good birth outcomes were significantly lower in the day-7 group, whereas rates of total pregnancy loss and clinical pregnancy loss were significantly higher. Conversely, among those receiving late blastocyst transfer (stage 5–6), the live birth rate was significantly higher in the day-7 group (50.2% vs. 36.5%; absolute difference, 13.75%; 95% CI, 6.57% to 20.93%; *P* < 0.001). Likewise, rates of clinical pregnancy and good birth outcomes were significantly higher in the day-7 group, while rates of total pregnancy loss, biochemical pregnancy loss, and clinical pregnancy loss were significantly lower (**Table 3**). In multivariable analyzes, after adjustment for confounders, day-7 (vs. day-6) progesterone exposure was negatively associated with LBR among early blastocysts (adjusted OR = 0.70; 95% CI, 0.55-0.87; *P* = 0.002), and positively associated with LBR among late blastocysts (adjusted OR = 1.82; 95% CI, 1.34-2.48; *P* < 0.001) (**Table 4**).

**Table 3:** Pregnancy and perinatal outcomes stratified by blastocyst expansion stage in day-6 versus day-7 groups.

Outcomes	Day-6 progesterone group	Day-7 progesterone group	Absolute difference	Odds ratio	*P* value
			(95% CI)	(95% CI)	
Early blastocyst (stage 3-4)	N=571	N=754			
Live birth	264/571 (46.2)	281/754 (37.3)	-8.97 (-14.32 to -3.62)	0.69 (0.55 to 0.86)	0.001
Biochemical pregnancy	368/571 (64.4)	430/754 (57.0)	-7.42 (-12.70 to -2.14)	0.73 (0.59 to 0.92)	0.006
Clinical pregnancy	324/571 (56.7)	370/754 (49.1)	-7.67 (-13.08 to -2.26)	0.73 (0.59 to 0.91)	0.006
Total pregnancy loss	98/368 (26.6)	146/430 (34.0)	7.32 (0.96 to 13.68)	1.42 (1.04 to 1.92)	0.025
Biochemical pregnancy loss	44/368 (12.0)	60/430 (14.0)	2.00 (-2.66 to 6.66)	1.19 (0.79 to 1.81)	0.404
Clinical pregnancy loss	54/324 (16.7)	86/370 (23.2)	6.58 (0.66 to 12.49)	1.51 (1.04 to 2.21)	0.031
Ectopic pregnancy	5/324 (1.5)	3/370 (0.8)	-0.73 (-2.36 to 0.89)	0.52 (0.12 to 2.20)	0.483
Good birth outcome	228/571 (39.9)	238/754 (31.6)	-8.36 (-13.57 to -3.16)	0.69 (0.55 to 0.87)	0.002
Gestational age*	38.2 (1.7)	38.3 (1.7)	0.1 (-0.1 to 0.3)		0.554
Preterm birth*	26/264 (9.8)	30/281 (10.7)	0.83 (-4.27 to 5.92)	1.09 (0.63 to 1.90)	0.75
Cesarean section*	179/264 (67.8)	188/281 (66.9)	-0.90 (-8.78 to 6.98)	0.96 (0.67 to 1.37)	0.823
Hypertensive disorders of pregnancy*	20/264 (7.6)	21/281 (7.5)	-0.10 (-4.53 to 4.33)	0.99 (0.52 to 1.86)	0.964
Gestational diabetes*	76/264 (28.8)	75/281 (26.7)	-2.10 (-9.62 to 5.42)	0.90 (0.62 to 1.31)	0.585
Major congenital anomaly*	1/264 (0.4)	5/281 (1.8)	1.40 (-0.31 to 3.11)	4.76 (0.55 to 41.05)	0.218
Birthweight (g)†	3189 (522)	3178 (488)	-11 (-85 to 63)		0.800
Low birthweight†	15/263 (5.7)	21/281 (7.5)	1.77 (-2.39 to 5.93)	1.34 (0.67 to 2.65)	0.407
Macrosomia†	7/263 (2.7)	7/281 (2.5)	-0.17 (-2.84 to 2.50)	0.93 (0.32 to 2.70)	0.900
Small for gestational age†	16/263 (6.1)	31/281 (11.0)	4.95 (0.28 to 9.61)	1.91 (1.02 to 3.59)	0.040
Large for gestational age†	23/263 (8.7)	28/281 (10.0)	1.22 (-3.67 to 6.11)	1.15 (0.65 to 2.06)	0.626
Stillbirth	0	0			
Late blastocyst (stage 5-6)	N=307	N=426			
Live birth	112/307 (36.5)	214/426 (50.2)	13.75 (6.57 to 20.93)	1.76 (1.30 to 2.37)	<0.001
Biochemical pregnancy	182/307 (59.3)	280/426 (65.7)	6.44 (-0.66 to 13.55)	1.32 (0.97 to 1.78)	0.075
Clinical pregnancy	154/307 (50.2)	254/426 (59.6)	9.46 (2.18 to 16.74)	1.47 (1.09 to 1.97)	0.011
Total pregnancy loss	67/182 (36.8)	63/280 (22.5)	-14.31 (-22.86 to -5.77)	0.50 (0.33 to 0.75)	0.001
Biochemical pregnancy loss	28/182 (15.4)	26/280 (9.3)	-6.10 (-12.35 to 0.15)	0.56 (0.32 to 1.00)	0.046
Clinical pregnancy loss	39/154 (25.3)	37/254 (14.6)	-10.76 (-18.88 to -2.63)	0.50 (0.30 to 0.83)	0.007
Ectopic pregnancy	1/154 (0.6)	1/254 (0.4)	-0.26 (-1.74 to 1.23)	0.60 (0.04 to 9.74)	1.000
Good birth outcome	88/307 (28.7)	170/426 (39.9)	11.24 (4.37 to 18.11)	1.65 (1.21 to 2.26)	0.002
Gestational age*	38.1 (1.9)	38.1 (2.0)	0.0 (-0.4 to 0.4)		0.938
Preterm birth*	13/112 (11.6)	32/214 (15.0)	3.35 (-4.27 to 10.96)	1.34 (0.67 to 2.67)	0.406
Cesarean section*	78/112 (69.6)	154/214 (72.0)	2.32 (-8.11 to 12.75)	1.12 (0.68 to 1.85)	0.661
Hypertensive disorders of pregnancy*	8/112 (7.1)	17/214 (7.9)	0.80 (-5.19 to 6.79)	1.12 (0.47 to 2.69)	0.796
Gestational diabetes*	25/112 (22.3)	60/214 (28.0)	5.72 (-4.07 to 15.50)	1.36 (0.79 to 2.32)	0.264
Major congenital anomaly*	1/112 (0.9)	1/214 (0.5)	-0.43 (-2.39 to 1.54)	0.52 (0.03 to 8.41)	1.000
Birthweight (g)†	3134 (524)	3138 (530)	4 (-116 to 124)		0.937
Low birthweight†	12/112 (10.7)	19/212 (9.0)	-1.75 (-8.65 to 5.15)	0.82 (0.38 to 1.76)	0.610
Macrosomia†	6/112 (5.4)	9/212 (4.2)	-1.11 (-6.09 to 3.86)	0.78 (0.27 to 2.26)	0.651
Small for gestational age†	11/112 (9.8)	20/212 (9.5)	-0.39 (-7.16 to 6.38)	0.96 (0.44 to 2.09)	0.921
Large for gestational age†	10/112 (8.9)	19/212 (9.0)	0.03 (-6.50 to 6.57)	1.01 (0.45 to 2.25)	0.982
Stillbirth	2/114 (1.8)	0/214	-1.75 (-4.16 to 0.66)		0.120

Data are n (%) or mean (SD). *Among live newborns. †Among all singletons.

**Table 4:** Multivariable logistic regression analysis of live birth in blastocyst expansion stage subgroups.

Parameters	Early blastocysts (n=1325)		Late blastocysts (n=733)	
	Adjusted OR (95%CI)	*P* value	Adjusted OR (95%CI)	*P* value
Age at retrieval (years)	0.96 (0.93-0.98)	0.001	0.99 (0.95-1.03)	0.527
PGT cycle (yes vs. no)	2.10 (1.49-2.94)	< 0.001	1.29 (0.88-1.90)	0.195
Body mass index (kg/m2)	0.97 (0.93-1.00)	0.085	0.99 (0.95-1.04)	0.700
Number of oocytes retrieved	1.00 (0.99-1.02)	0.977	1.02 (1.00-1.04)	0.070
Endometrial thickness	1.02 (0.96-1.08)	0.569	0.99 (0.92-1.08)	0.872
Regimens (GnRH-agonist HRT vs. HRT)	0.95 (0.73-1.23)	0.693	0.93 (0.64-1.34)	0.686
Indications for IVF		0.238		0.271
Tubal factor	Reference		Reference	
Male factor	1.29 (0.93-1.79)	0.123	1.34 (0.79-2.28)	0.279
Others	1.12 (0.79-1.57)	0.520	1.09 (0.74-1.63)	0.654
Combined factors	0.79 (0.53-1.18)	0.242	1.83 (1.03-3.25)	0.041
Unexplained	1.10 (0.66-1.82)	0.725	1.34 (0.65-2.77)	0.427
Number of IVF cycles		0.611		0.096
1	Reference		Reference	
2	1.12 (0.81-1.54)	0.501	1.44 (0.92-2.24)	0.109
≥3	0.81 (0.43-1.52)	0.502	0.60 (0.28-1.29)	0.191
Progesterone exposure days (7 vs. 6)	0.70 (0.55-0.87)	0.002	1.82 (1.34-2.48)	< 0.001
Morphological quality grades		0.023		0.044
Grade 1	1.41 (0.92-2.18)	0.117	2.24 (0.99-5.08)	0.053
Grade 2	1.37 (1.08-1.74)	0.010	1.39 (1.01-1.92)	0.044
Grade 3	Reference		Reference	
Progesterone supplementation (Intramuscular vs vaginal)	0.94 (0.75-1.19)	0.627	0.91 (0.67-1.24)	0.538

OR, odds ratio; IVF, *in-vitro* fertilization; PGT, preimplantation genetic testing; HRT, hormone replacement therapy.

Models were adjusted for age at retrieval, body mass index, IVF indication, number of IVF cycles, number of oocytes retrieved, PGT cycle, cycle regimen, endometrial thickness, route of progesterone administration, morphological quality grade, and duration of progesterone exposure.

Morphological quality grades are defined by the combination of inner cell mass (ICM) and trophectoderm (TE) scores: Grade 1 (AA, AB, BA); Grade 2 (BB); and Grade 3 (BC, CB, AC, CA).

Pregnancy outcomes were further evaluated separately in patients undergoing HRT cycles and GnRH-agonist HRT cycles, with comparable results between the day-6 and day-7 progesterone exposure groups in both regimens (**Supplementary Tables 5**, **6**). Subgroup analyzes within each cycle regimen revealed a significant interaction between blastocyst expansion stage and progesterone exposure duration, with significant differences in pregnancy outcomes between the day-6 and day-7 progesterone groups for both early- and late-stage blastocysts. These findings indicate that the effect of progesterone exposure timing on pregnancy outcomes in day-6 blastocyst transfer is consistent across both endometrial preparation regimens.

## Discussion

This study found no significant difference in overall live birth rates between day-6 and day-7 progesterone exposure for day-6 blastocyst transfers in artificial FET cycles. Importantly, this is the first study to identify a significant interaction between blastocyst expansion stage and the duration of progesterone exposure. Subgroup analyzes further demonstrated that early-stage blastocysts (stages 3–4) achieved significantly higher live birth rates with day-6 progesterone exposure, whereas late-stage blastocysts (stages 5–6) benefited from day-7 exposure.

The optimal duration of progesterone supplementation before frozen–thawed embryo transfer remains controversial, and the ideal timing of blastocyst transfer following progesterone priming in artificial cycles is poorly defined. Roelens et al. reported comparable overall live birth rates between blastocyst transfers performed on day 6 versus day 7 of progesterone exposure; however, among day-6 blastocysts, transfers on day 6 were associated with a significantly higher miscarriage rate, while outcomes for day-5 blastocysts were similar between regimens (16). Conversely, another retrospective study reported higher LBRs for blastocyst transfer on day 6 compared with day 7 (28); however, these findings are limited by substantial population heterogeneity and inadequate statistical adjustment, compromising result reliability. Only one RCT has examined the duration of progesterone exposure before blastocyst transfer, reporting no significant difference in clinical pregnancy rate between the day-5 and day-7 regimens (15); however, its limited sample size, inclusion of double embryo transfers, and lack of stratification by blastocyst formation day may have introduced bias.

Successful implantation relies on precise synchronization between embryo development and endometrial receptivity (6). Although early studies suggested a relatively wide implantation window following progesterone exposure, emerging evidence indicates that the WOI is narrower than previously recognized (10, 11). Suboptimal endometrial priming by progesterone may disrupt the delicate crosstalk between the embryo and the endometrium, resulting in compromised implantation and adverse pregnancy outcomes (8, 9). These findings underscore the importance of achieving optimal alignment between embryo developmental stage and endometrial receptivity.

Accumulating evidence shows that embryos reaching the blastocyst stage on day 6 exhibit lower implantation rates than those reaching this stage on day 5 (19–23). This difference has traditionally been attributed to reduced intrinsic embryonic competence (20, 22). However, embryo–endometrium dyssynchrony represents an alternative and underexplored explanation. Importantly, most prior studies comparing day-5 and day-6 blastocyst transfers scheduled embryo transfer uniformly on the sixth day of progesterone exposure, irrespective of developmental timing or expansion stage. Even when embryo quality and endometrial receptivity are individually adequate, implantation may still fail if their temporal alignment is suboptimal. Previous studies have shown that both blastulation speed and blastocyst expansion grade are independent predictors of live birth rate (17, 24).

Day-6 blastocysts therefore represent a unique clinical challenge: delayed blastulation combined with heterogeneous expansion stages may interact with progesterone exposure duration to influence implantation success. Few studies have specifically addressed the optimal progesterone exposure duration for day-6 blastocysts in FET, and results remain conflicting. Existing evidence can be broadly categorized into three groups: some have reported superior pregnancy outcomes with day-7 versus day-6 progesterone exposure (16, 18); others have found the opposite (29, 30); while several studies have shown no significant difference between the two regimens (31, 32). Critically, several studies failed to distinguish between natural and artificial cycles, and none accounted for blastocyst expansion stage. Our findings support this concept by demonstrating stage-specific responses to progesterone timing. From a biological perspective, we hypothesize that early-stage blastocysts (stages 3-4) might require additional time *in utero* to complete expansion and hatch from the zona pellucida. Therefore, initiating transfer on day 6 of progesterone exposure could potentially provide a temporal buffer, allowing the embryo to hatch concurrently as the endometrium reaches peak receptivity. Conversely, because late-stage blastocysts (stages 5-6) are actively herniating or fully hatched, they are theoretically more prepared for immediate implantation. Transferring these advanced embryos on day 7 might better synchronize their invasive potential with a more mature secretory endometrium. The opposing effects of progesterone duration on early- versus late-stage blastocysts likely explain why overall live birth rates were comparable between day-6 and day-7 progesterone regimens in both previous studies and the present cohort. Although day-5 blastocysts were explicitly excluded from our formal analysis, a preliminary assessment of their pregnancy outcomes during the same study period (data not shown) indicated a higher live birth rate following day-6 progesterone exposure compared with day-7 exposure. This observation must be interpreted with caution and may be partially attributable to the extremely low proportion of late-stage embryos among day-5 blastocysts (0.8%). Consequently, these unanalyzed data are presented strictly as a hypothesis-generating point. Future dedicated studies are warranted to comprehensively evaluate whether the conventional day-6 progesterone regimen remains optimal for day-5 blastocyst transfers.

Most prior investigations of the optimal window of implantation have evaluated endometrial advancement, progesterone duration, and blastocyst developmental timing as independent factors. Our results highlight the importance of their dynamic interplay, particularly for day-6 blastocysts. These findings challenge the conventional practice of applying a uniform progesterone duration regardless of blastocyst formation day or expansion stage and underscore the potential value of individualized transfer strategies. They also point to potential limitations of current endometrial receptivity assessment approaches, which may not fully capture embryo-specific developmental dynamics.

Several limitations should be acknowledged. First, the retrospective design and non-randomized allocation of progesterone exposure duration inherently introduce the risk of selection bias. Although the choice of a day-6 versus day-7 transfer was primarily driven by non-clinical factors (physician habits and patient convenience) rather than clinical indications—a premise supported by the highly balanced baseline characteristics between groups—unmeasured residual confounding cannot be entirely ruled out. Specifically, our retrospective dataset could not fully capture potential unmeasured confounders, such as variations in luteal phase support protocols, the differing experience levels of individual physicians and embryologists, and the technical details of the vitrification and warming processes. These unmeasured factors could potentially exert an independent influence on clinical outcomes. Second, the stringent exclusion criteria (e.g., maternal age > 40 years, RIF, RPL, and thin endometrium) yielded a study population with a relatively favorable prognosis, which inherently limits the external validity of our findings. Because these challenging clinical populations typically harbor complex and intertwined mechanisms of implantation failure (such as profoundly impaired endometrial receptivity or intrinsically high embryonic aneuploidy rates), exactly how the interaction between progesterone duration and blastocyst expansion stage manifests in these poor-prognosis groups remains unclear. Therefore, our findings cannot be readily extrapolated to these specific cohorts and necessitate further targeted exploration in future studies. Third, patients undergoing PGT were not excluded; although PGT proportions were balanced and no significant interaction with progesterone exposure was detected, residual confounding cannot be entirely excluded. Accordingly, these findings should be interpreted with caution.

In conclusion, among women undergoing day-6 blastocyst transfer in artificial FET cycles, overall live birth rates were comparable between transfers performed on day 6 and day 7 of progesterone supplementation. However, progesterone timing interacted significantly with blastocyst expansion stage: early-stage blastocysts achieved higher live birth rates with day-6 exposure, whereas late-stage blastocysts benefited from day-7 exposure. These findings support a more individualized approach to progesterone timing based on blastocyst developmental characteristics. Well-designed prospective studies are warranted to confirm the optimal duration of progesterone exposure for blastocysts at different expansion stages.

## Data Availability

The raw data supporting the conclusions of this article will be made available by the authors, without undue reservation.
