# Supplementary material for: Interaction between progesterone exposure duration and blastocyst expansion stage on live birth following frozen–thawed transfer of day-6 blastocysts
Source: Front Endocrinol (Lausanne). 2026 Apr 23;17:1813202. doi: 10.3389/fendo.2026.1813202 (PMC13149102; doi:10.3389/fendo.2026.1813202)
Supplement: Supplementary file 1 [file Table1.docx]

**Table S1.** Subgroup analyses of baseline and cycle characteristics stratified by blastocyst expansion stage in day-6 versus day-7 groups.

| **Parameters** | **Early blastocyst (stage 3-4)** | | | | | | | | **Late blastocyst (stage 5-6)** | | | | |  |  |
| --- | --- | --- | --- | --- | --- | --- | --- | --- | --- | --- | --- | --- | --- | --- | --- |
|  | **Day-6 progesterone**  **(n = 571)** | | **Day-7 progesterone**  **(n = 754)** | | | ***P*** | **Day-6 progesterone**  **(n = 307)** | | | | **Day-7 progesterone**  **(n = 426)** | | ***P*** |  |  |
| Age at retrieval (years)  Age at embryo transfer (years)  Body mass index (kg/m2)  Duration of infertility (years)  Type of infertility  Primary  Secondary  Indications for IVF  Tubal factor  Male factor  Others  Combined factors  Unexplained  Antral follicle count  Number of IVF cycles†  1  2  ≥3  ICSI treatment  No. of oocytes retrieved  PGT cycle  PGT method  PGT-A  PGT-M  PGT-SR  Embryo transfer cycle rank†  1  2  3  4  Endometrial thickness*  Good-quality blastocyst transfer  Morphological quality grades  Grade 1  Grade 2  Grade 3  Cycle regimens  HRT  GnRH-agonist HRT  Progesterone supplementation  Intramuscular + oral  Vaginal + oral | | 31 (28, 35)  33 (30, 36)  21.5 (19.6, 23.5)  3.0 (1.0, 4.0)  307 (53.8)  264 (46.2)  222 (38.9)  109 (19.1)  155 (27.1)  61 (10.7)  24 (4.2)  15 (10, 20)  462 (80.9)  91 (15.9)  18 (3.2)  282 (49.4)  16 (11, 22)  128 (22.4)  41(32.0)  41 (32.0)  46 (35.9)  192 (33.6)  210 (36.8)  115 (20.1)  54 (9.5)  9.0 (8.0, 11.0)  285 (49.9)  53 (9.3)  232 (40.6)  286 (50.1)  422 (73.9)  149 (26.1)  246 (43.1)  325 (56.9) | | 32 (29, 35)  33 (30, 36)  21.3 (19.5, 23.3)  3.0 (1.0, 5.0)  385 (51.1)  369 (48.9)  301 (39.9)  114 (15.1)  200 (26.5)  87 (11.5)  52 (6.9)  16 (10, 20)  624 (82.8)  102 (13.5)  28 (3.7)  348 (46.2)  15 (11, 21)  167 (22.1)  50 (29.9)  55 (32.9)  62 (37.1)  264 (35.0)  266 (35.3)  157 (20.8)  67 (8.9)  9.0 (8.0, 10.5)  356 (47.2)  48 (6.4)  308 (40.8)  398 (52.8)  561 (74.4)  193 (25.6)  293 (38.9)  461 (61.1) | 0.467  0.808  0.086  0.213  0.329  0.112  0.510  0.422  0.243  0.201  0.907  0.928  0.902  0.241  0.331  0.130  0.838  0.121 | | | 31 (28, 34)  32 (29, 35)  21.1 (19.6, 24.0)  2.0 (1.0, 4.0)  135 (44.0)  172 (56.0)  130 (42.3)  33 (10.7)  100 (32.6)  26 (8.5)  18 (5.9)  16 (10, 22)  254 (82.7)  39 (12.7)  14 (4.6)  132 (43.0)  17 (12, 23)  91 (29.6)  33 (36.3)  26 (28.6)  32 (35.2)  140 (45.6)  98 (31.9)  54 (17.6)  15 (4.9)  9.0 (8.0, 10.5)  192 (62.5)  14 (4.6)  178 (58.0)  115 (37.5)  234 (76.2)  73 (23.8)  128 (41.7)  179 (58.3) | | 32 (28, 35)  32 (30, 36)  21.7 (19.9, 23.6)  2.0 (1.0, 4.0)  195 (45.8)  231 (54.2)  159 (37.3)  41 (9.6)  169 (39.7)  39 (9.2)  18 (4.2)  15 (10, 22)  348 (81.7)  59 (13.8)  19 (4.5)  177 (41.5)  16 (11, 22)  138 (32.4)  38 (27.5)  44 (31.9)  56 (40.6)  186 (43.7)  142 (33.3)  73 (17.1)  25 (5.9)  9.0 (8.0, 11.0)  262 (61.5)  14 (3.3)  248 (58.2)  164 (38.5)  326 (76.5)  100 (23.5)  177 (41.5)  249 (58.5) | | 0.108  0.335  0.254  0.315  0.629  0.299  0.517  0.903  0.695  0.218  0.428  0.375  0.897  0.838  0.775  0.667  0.924  0.969 | |  | |

Data are median (IQR) or n (%). IVF, in-vitro fertilization; ICSI, intracytoplasmic sperm injection; FET, frozen embryo transfer; PGT, preimplantation genetic testing; A, aneuploidy; M, monogenic disorder; SR, chromosomal structural rearrangement; HRT, hormone replacement therapy. *The day of frozen embryo transfer. †These variables denote the ordinal position of the included cycle. Morphological quality grades are defined by the combination of inner cell mass (ICM) and trophectoderm (TE) scores: Grade 1 (AA, AB, BA); Grade 2 (BB); and Grade 3 (BC, CB, AC, CA).

**Table S2.** Live birth rates stratified by four blastocyst expansion stages in day-6 versus day-7 groups.

| **Subgroup** | **Day-6 progesterone group** | **Day-7 progesterone group** | **Odds Ratio**  **(95% CI)** | ***P* value** | ***P* value for interaction** |
| --- | --- | --- | --- | --- | --- |
| **Blastocyst expansion stage**  Stage 3  Stage 4  Stage 5  Stage 6 | 18/41 (43.9)  246/530 (46.4)  52/171 (30.4)  60/136 (44.1) | 10/49 (20.4)  271/705 (38.4)  114/235 (48.5)  100/191 (52.4) | 0.328 (0.129 to 0.830)  0.721 (0.574 to 0.906)  2.156 (1.425 to 3.263)  1.392 (0.895 to 2.165) | 0.016  0.005  < 0.001  0.142 | < 0.001 |

**Table S3.** Univariable logistic regression analysis of live birth in all patients.

| **Parameters** | **Unadjusted OR** | **95% CI** | ***P*-value** |
| --- | --- | --- | --- |
| Age at retrieval (years) | 0.965 | 0.945-0.985 | 0.001 |
| Body mass index (kg/m^2^)  Duration of infertility (years)  Secondary vs primary infertility  Indications for IVF  Tubal factor  Male factor  Others  Combined factors  Unexplained | 0.980  0.945  0.927  Reference  1.377  1.582  1.242  1.183 | 0.953-1.008  0.914-0.977  0.779-1.105  1.052-1.802  1.280-1.956  0.914-1.688  0.791-1.770 | 0.152  0.001  0.398  0.001  0.020  < 0.001  0.166  0.413 |
| Antral follicle count  Number of IVF cycles  1  2  ≥3  ICSI treatment | 1.009  Reference  1.258  0.766  1.551 | 0.999-1.019  0.980-1.614  0.478-1.226  1.300-1.849 | 0.089  0.090  0.072  0.266  < 0.001 |
| Number of oocytes retrieved  PGT cycle (yes vs no)  Embryo transfer cycle rank  1  2  3  4  Endometrial thickness*  Good-quality blastocyst transfer (yes vs no)  Morphological quality grades  Grade 1  Grade 2  Grade 3  Regimens (GnRH-agonist HRT vs. HRT)  Progesterone supplementation  (Intramuscular vs vaginal)  Blastocyst expansion stage (late vs. early)  Progesterone exposure days (7 vs. 6) | 1.015  1.793  Reference  0.922  0.921  0.898  1.007  1.502  1.671  1.480  Reference  0.871  0.938  1.146  0.965 | 1.004-1.026  1.468-2.190  0.751-1.132  0.722-1.176  0.637-1.268  0.963-1.054  1.259-1.792  1.156-2.417  1.234-1.776  0.711-1.067  0.785-1.120  0.955-1.375  0.808-1.151 | 0.008  < 0.001  0.832  0.438  0.511  0.542  0.749  < 0.001  < 0.001  0.006  < 0.001  0.182  0.479  0.142  0.695 |

OR, odds ratio; IVF, in-vitro fertilization; ICSI, intracytoplasmic sperm injection; FET, frozen embryo transfer; PGT, preimplantation genetic testing; HRT, hormone replacement therapy. *The day of frozen embryo transfer.

Morphological quality grades are defined by the combination of inner cell mass (ICM) and trophectoderm (TE) scores: Grade 1 (AA, AB, BA); Grade 2 (BB); and Grade 3 (BC, CB, AC, CA).

**Table S4. Multivariable logistic regression analysis of live birth in all patients.**

|  | **Model 1** | | | | **Model 2** | | | | |
| --- | --- | --- | --- | --- | --- | --- | --- | --- | --- |
| **Parameters** | **Adjusted OR (95%CI)** | | | ***P* value** | | **Adjusted OR (95%CI)** | | ***P* value** |  |
| Age at retrieval (years)  PGT cycle (yes vs. no)  Body mass index (kg/m^2^)  Number of oocytes retrieved  Endometrial thickness  Regimens (GnRH-agonist HRT vs. HRT)  Indications for IVF  Tubal factor  Male factor  Others  Combined factors  Unexplained  Number of IVF cycles  1  2  ≥3  Morphological quality grades  Grade 1  Grade 2  Grade 3  Progesterone supplementation  (Intramuscular vs vaginal)  Blastocyst expansion stage (late vs. early)  Progesterone exposure days (7 vs. 6)  Interaction term | | 0.97 (0.95-0.99)  1.73 (1.35-2.22)  0.98 (0.95-1.01)  1.01 (0.99-1.02)  1.01 (0.97-1.06)  0.94 (0.76-1.16)  Reference  1.33 (1.01-1.75)  1.13 (0.88-1.46)  1.04 (0.76-1.44)  1.13 (0.75-1.70)  Reference  1.21 (0.94-1.56)  0.73 (0.45-1.17)  1.59 (1.09-2.32)  1.35 (1.12-1.63)  Reference  0.92 (0.77-1.11)  1.06 (0.88-1.29)  0.99 (0.82-1.18) | 0.003  < 0.001  0.206  0.280  0.580  0.574  0.357  0.044  0.340  0.802  0.563  0.121  0.141  0.191  0.002  0.015  0.002  0.378  0.532  0.873 | | | 0.97 (0.94 to 0.99)  1.75 (1.36 to 2.26)  0.98 (0.95-1.01)  1.01 (0.99-1.02)  1.01 (0.97-1.06)  0.94 (0.76-1.16)  Reference  1.31 (0.99-1.72)  1.11 (0.86-1.43)  1.03 (0.75-1.43)  1.17 (0.78-1.77)  Reference  1.20 (0.93-1.55)  0.73 (0.45-1.18)  1.57 (1.07-2.29)  1.36 (1.12-1.64)  Reference  0.93 (0.77-1.11)  0.61 (0.46-0.82)  0.70 (0.56-0.88)  2.58 (1.76-3.77) | 0.002  < 0.001  0.143  0.273  0.608  0.568  0.420  0.059  0.438  0.835  0.454  0.147  0.169  0.201  0.002  0.020  0.002  0.421  0.001  0.002  < 0.001 | |  |

OR, odds ratio; IVF, in-vitro fertilization; PGT, preimplantation genetic testing; HRT, hormone replacement therapy.

Model 1 were adjusted for age, body mass index, IVF indication, number of IVF cycles, number of oocytes retrieved, PGT cycle, cycle regimen, endometrial thickness, route of progesterone administration, morphological quality grade, blastocyst expansion stage, and progesterone exposure days. Model 2 was further adjusted for an interaction term between blastocyst expansion stage and progesterone exposure days in addition to the covariates included in Model 1.

Morphological quality grades are defined by the combination of inner cell mass (ICM) and trophectoderm (TE) scores: Grade 1 (AA, AB, BA); Grade 2 (BB); and Grade 3 (BC, CB, AC, CA).

**Table S5.** Pregnancy outcomes in patients with HRT cycles in day-6 versus day-7 groups.

| **Outcomes** | **Day-6 progesterone group** | **Day-7 progesterone group** | **Absolute difference**  **(95% CI)** | **Odds ratio**  **(95% CI)** | ***P* value** |
| --- | --- | --- | --- | --- | --- |
| **Patients with HRT cycles**  Live birth  Biochemical pregnancy  Clinical pregnancy  Total pregnancy loss  Biochemical pregnancy loss  Clinical pregnancy loss  Ectopic pregnancy  Good birth outcome  **Early blastocyst (stage 3-4)**  Live birth | N=656  283/656 (43.1)  412/656 (62.8)  360/656 (54.9)  123/412 (29.9)  52/412 (12.6)  71/360 (19.7)  5/360 (1.4)  242/656 (36.9)  N=422  195/422 (46.2) | N=887  383/887 (43.2)  530/887 (59.8)  472/887 (53.2)  141/530 (26.6)  58/530 (10.9)  83/472 (17.6)  4/472 (0.8)  326/887 (36.8)  N=561  217/561 (38.7) | 0.04 (-4.96 to 5.04)  -3.05 (-7.96 to 1.86)  -1.66 (-6.69 to 3.36)  -3.25 (-9.05 to 2.55)  -1.68 (-5.84 to 2.49)  -2.14 (-7.49 to 3.22)  -0.54 (-2.01 to 0.92)  -0.14 (-5.01 to 4.73)  -7.53 (-13.76 to -1.29) | 1.00 (0.82 to 1.23)  0.88 (0.71 to 1.08)  0.94 (0.76 to 1.15)  0.85 (0.64 to 1.13)  0.85 (0.57 to 1.27)  0.87 (0.61 to 1.23)  0.61 (0.16 to 2.28)  0.99 (0.81 to 1.23)  0.73 (0.57 to 0.95) | 0.988  0.224  0.517  0.270  0.426  0.432  0.511  0.956  0.018 |
| Biochemical pregnancy  Clinical pregnancy  Total pregnancy loss  Biochemical pregnancy loss  Clinical pregnancy loss  Ectopic pregnancy  Good birth outcome  **Late blastocyst (stage 5-6)**  Live birth  Biochemical pregnancy  Clinical pregnancy  Total pregnancy loss  Biochemical pregnancy loss  Clinical pregnancy loss  Ectopic pregnancy  Good birth outcome | 272/422 (64.5)  242/422 (57.3)  72/272 (26.5)  30/272 (11.0)  42/242 (17.4)  5/242 (2.1)  169/422 (40.0)  N=234  88/234 (37.6)  140/234 (59.8)  118/234 (50.4)  51/140 (36.4)  22/140 (15.7)  29/118 (24.6)  0/118  73/234 (31.2) | 317/561 (56.5)  278/561 (49.6)  97/317 (30.6)  39/317 (12.3)  58/278 (20.9)  3/278 (1.1)  189/561 (33.7)  N=326  166/326 (50.9)  213/326 (65.3)  194/326 (59.5)  44/213 (20.7)  19/213 (8.9)  25/194 (12.9)  1/194 (0.5)  137/326 (42.0) | -7.95 (-14.09 to -1.81)  -7.79 (-14.07 to -1.52)  4.13 (-3.17 to 11.42)  1.27 (-3.92 to 6.46)  3.51 (-3.24 to 10.26)  -0.99 (-3.15 to 1.18)  -6.36 (-12.45 to -0.26)  13.31 (5.07 to 21.56)  5.51 (-2.62 to 13.64)  9.08 (0.75 to 17.41)  -15.77 (-25.42 to -6.12)  -6.79 (-13.94 to 0.35)  -11.69 (-20.78 to -2.60)  0.52 (-0.49 to 1.52)  10.83 (2.83 to 18.82) | 0.72 (0.55 to 0.93)  0.73 (0.57 to 0.94)  1.22 (0.85 to 1.76)  1.13 (0.68 to 1.88)  1.26 (0.81 to 1.95)  0.52 (0.12 to 2.19)  0.76 (0.59 to 0.99)  1.72 (1.22 to 2.42)  1.27 (0.89 to 1.79)  1.44 (1.03 to 2.03)  0.45 (0.28 to 0.73)  0.53 (0.27 to 1.01)  0.45 (0.25 to 0.82)  1.60 (1.12 to 2.28) | 0.012  0.015  0.269  0.632  0.311  0.482  0.040  0.002  0.183  0.033  0.001  0.051  0.008  1.000  0.009 |

**Table S6.** Pregnancy outcomes in patients with GnRH-agonist HRT cycles in day-6 versus day-7 groups.

| **Outcomes** | **Day-6 progesterone group** | **Day-7 progesterone group** | **Absolute difference**  **(95% CI)** | **Odds ratio**  **(95% CI)** | ***P* value** |
| --- | --- | --- | --- | --- | --- |
| **Patients with GnRH-α HRT cycles**  Live birth  Biochemical pregnancy  Clinical pregnancy  Total pregnancy loss  Biochemical pregnancy loss  Clinical pregnancy loss  Ectopic pregnancy  Good birth outcome  **Early blastocyst (stage 3-4)**  Live birth | N=222  93/222 (41.9)  138/222 (62.2)  118/222 (53.2)  42/138 (30.4)  20/138 (14.5)  22/118 (18.6)  1/118 (0.8)  74/222 (33.3)  N=149  69/149 (46.3) | N=293  112/293 (38.2)  180/293 (61.4)  152/293 (51.9)  68/180 (37.8)  28/180 (15.6)  40/152 (26.3)  0/152  82/293 (28.0)  N=193  64/193 (33.2) | -3.67 (-12.22 to 4.88)  -0.73 (-9.20 to 7.74)  -1.28 (-9.98 to 7.43)  7.34 (-3.10 to 17.79)  1.06 (-6.84 to 8.97)  7.67 (-2.25 to 17.59)  -0.85 (-2.50 to 0.81)  -5.35 (-13.40 to 2.71)  -13.15 (-23.55 to -2.75) | 0.86 (0.60 to 1.23)  0.97 (0.68 to 1.39)  0.95 (0.67 to 1.35)  1.39 (0.87 to 2.22)  1.09 (0.58 to 2.02)  1.56 (0.87 to 2.80)  0.78 (0.53 to 1.13)  0.58 (0.37 to 0.89) | 0.400  0.866  0.774  0.172  0.793  0.137  0.437  0.191  0.013 |
| Biochemical pregnancy  Clinical pregnancy  Total pregnancy loss  Biochemical pregnancy loss  Clinical pregnancy loss  Ectopic pregnancy  Good birth outcome  **Late blastocyst (stage 5-6)**  Live birth  Biochemical pregnancy  Clinical pregnancy  Total pregnancy loss  Biochemical pregnancy loss  Clinical pregnancy loss  Ectopic pregnancy  Good birth outcome | 96/149 (64.4)  82/149 (55.0)  26/96 (27.1)  14/96 (14.6)  12/82 (14.6)  0/82  59/149 (39.6)  N=73  24/73 (32.9)  42/73 (57.5)  36/73 (49.3)  16/42 (38.1)  6/42 (14.3)  10/36 (27.8)  1/36 (2.8)  15/73 (20.5) | 113/193 (58.5)  92/193 (47.7)  49/113 (43.4)  21/113 (18.6)  28/92 (30.4)  0/92  49/193 (25.4)  N=100  48/100 (48.0)  67/100 (67.0)  60/100 (60.0)  19/67 (28.4)  7/67 (10.4)  12/60 (20.0)  0/60  33/100 (33.0) | -5.88 (-16.24 to 4.48)  -7.37 (-18.02 to 3.29)  16.28 (3.53 to 29.03)  4.00 (-6.06 to 14.06)  15.80 (3.68 to 27.92)  /  -14.21 (-24.18 to -4.24)  15.12 (0.56 to 29.68)  9.47 (-5.15 to 24.08)  10.68 (-4.27 to 25.64)  -9.74 (-27.96 to 8.49)  -3.84 (-16.71 to 9.03)  -7.78 (-25.57 to 10.01)  -2.78 (-8.15 to 2.59)  12.45 (-0.62 to 25.52) | 0.78 (0.50 to 1.21)  0.74 (0.48 to 1.14)  2.06 (1.15 to 3.70)  1.34 (0.64 to 2.80)  2.55 (1.20 to 5.44)  /  0.52 (0.33 to 0.82)  1.88 (1.01 to 3.53)  1.50 (0.80 to 2.80)  1.54 (0.84 to 2.83)  0.64 (0.28 to 1.46)  0.70 (0.22 to 2.25)  0.65 (0.25 to 1.71)  /  1.90 (0.94 to 3.85) | 0.269  0.177  0.014  0.440  0.013  0.005  0.046  0.203  0.163  0.289  0.547  0.380  0.375  0.071 |

**Table S7.** Distribution of morphological quality grades across individual blastocyst expansion stages according to progesterone exposure duration.

| **Blastocyst expansion stage** | **Day-6 progesterone** | **Day-7 progesterone** | ***P*-value** |
| --- | --- | --- | --- |
| Stage 3 (n=90) |  |  | 0.208 |
| Grade 1  Grade 2  Grade 3  Stage 4 (n=1235) | 0  10 (24.4)  31 (75.6) | 0  18 (36.7)  31 (63.3) | 0.093 |
| Grade 1  Grade 2  Grade 3  Stage 5 (n=406)  Grade 1  Grade 2  Grade 3  Stage 6 (n=327)  Grade 1  Grade 2  Grade 3 | 53 (10.0)  222 (41.9)  255 (48.1)  12 (7.0)  84 (49.1)  75 (43.9)  2 (1.5)  94 (69.1)  40 (29.4) | 48 (6.8)  290 (41.1)  367 (52.1)  12 (5.1)  112 (47.7)  111 (47.2)  2 (1.0)  136 (71.2)  53 (27.7) | 0.637  0.886 |

Morphological quality grades are defined by the combination of inner cell mass (ICM) and trophectoderm (TE) scores: Grade 1 (AA, AB, BA); Grade 2 (BB); and Grade 3 (BC, CB, AC, CA).
